# Clinical Features of a Newly Described Mutation of Myelin Protein Zero in a Family

**DOI:** 10.7759/cureus.39884

**Published:** 2023-06-02

**Authors:** Vasudeva G Iyer, Lisa B Shields, Yi Ping Zhang, Christopher B Shields

**Affiliations:** 1 Neurodiagnostic Center of Louisville, Louisville, USA; 2 Norton Neuroscience Institute, Norton Healthcare, Louisville, USA; 3 Department of Neurological Surgery, University of Louisville School of Medicine, Louisville, USA

**Keywords:** charcot-marie-tooth disease, ultrasound, electromyography, myelin protein zero, neurology

## Abstract

Charcot-Marie-Tooth (CMT) disease is the most common hereditary neuropathy. Duplication of the *peripheral myelin protein-22* (*PMP22*) gene is the most frequent genetic abnormality in CMT disease. Although rare compared to *PMP22* gene mutations, many different *myelin protein zero* (*MPZ*) gene mutations have been described in patients with CMT disease. *MPZ* gene mutations are known to cause hereditary neuropathies with heterogenous phenotypes ranging from early-onset severe demyelinating to adult-onset axonal forms. MPZ, the major protein component of peripheral nerve myelin, is important for myelin compaction. We report a family in which a mother and her son, both with adult-onset CMT disease, showed a newly described mutation p.Glu37Lys of the *MPZ* gene. The clinical features of the mother provided insight into the progression of the disease over decades, while features in the early stage of the disease could be studied in the son. Clinical, electrodiagnostic, and sonographic findings are described in the early and late stages of the disease. The *MPZ* gene mutation p.Glu37Lys is associated with clinical features of a progressive axonal type of adult-onset CMT disease.

## Introduction

With a prevalence of one in 2,500 individuals, Charcot-Marie-Tooth (CMT) disease is the most common hereditary neuromuscular disorder marked by progressive distal muscle weakness, sensory loss, decreased or absent deep tendon reflexes (DTRs), and skeletal deformities such as pes cavus [1-4]. The autosomal dominant 1.5 Mb duplication of the *peripheral myelin protein-22* (*PMP22*) gene on chromosome 17p11.2 is identified in 70-80% of CMT cases (CMT1) [5]. Motor nerve conduction velocity (MNCV) distinguishes demyelinating CMT1 (MNCV is reduced with values <38 m/s) from axonal CMT2 (MNCV > 38 m/s) [6]. A high-resolution ultrasound may reveal diffuse enlargement of nerves over the length of the nerve [4].

While the *PMP22* gene mutation is most frequently confirmed in CMT disease, greater than 100 gene mutations are known to cause CMT phenotypes which may be inherited in an autosomal dominant, autosomal recessive, or X-linked manner [1,7]. A type I transmembrane protein consisting of 248 amino acids and a member of the immunoglobulin supergene family, myelin protein zero (MPZ), is the most abundant structural protein in the peripheral nervous system myelin [8,9]. Encoded by the *MPZ* gene and produced by Schwann cells, it is important for myelin compaction [8-10]. The MPZ protein serves as a double adhesion molecule with extracellular and cytoplasmic interactions that bind the myelin sheath [7]. *MPZ* gene mutations are being increasingly recognized as causes of certain forms of hereditary peripheral neuropathy including CMT disease and early-onset Dejerine-Sottas disease [9,10]. Affecting 4.1-5% of all CMT patients, MPZ mutations are associated with the autosomal dominant demyelinating neuropathy CMT1B and axonal neuropathy CMT2I/J [7,9]. Additionally, point mutations in the *MPZ* gene account for 10-12% of all dominant demyelinating CMT type 1 cases [10]. Although rare compared to *PMP22* gene mutations, there have been several recent publications on the genetic spectrum of CMT disease associated with the *MPZ* gene mutations [7-10]. 

In this report, we describe the clinical symptoms of two family members diagnosed with CMT2 disease with the MPZ genetic variant, one at the onset and the other in the late stage, both of whom underwent electrodiagnostic (EDX) and ultrasound (US) studies. The presenting symptoms, clinical and EDX findings, and US features are presented. The physiological role of the MPZ genetic variant and how missense mutations affects its function are discussed. 

## Case presentation

Two family members, a mother in the late stage and her son in the early stage of disease, underwent a focused neurological examination followed by EDX and US evaluation of peripheral nerves. The CMT neuropathy score (CMTNS) was estimated [2]. The EDX studies were done according to the protocols established in our facility [11]. The US study of the median and ulnar nerves was done with an 8-18 MHz probe and GE LOGIQ machine. Genetic tests were performed in an outside lab (Invitae Corporation, San Francisco, CA). This procedure involved the sequencing of genomic DNA by a hybridization-based protocol using Illumina technology. Reads were aligned to a reference sequence (GRCh37), and sequence changes were identified and interpreted in the context of a single clinically relevant transcript. Confirmational technologies included the following: Sanger sequencing, Pacific Biosciences SMRT sequencing, MLPA, MLPA-seq, and Array CGH. Informed consent was obtained from both patients. The Western IRB-Copernicus Group (WCG) IRB approved a request for a waiver of authorization for the use and disclosure of protected health information for this study. The review was conducted through an expedited process.

Patient 1 (mother)

Clinical Findings

**Figure 1:**
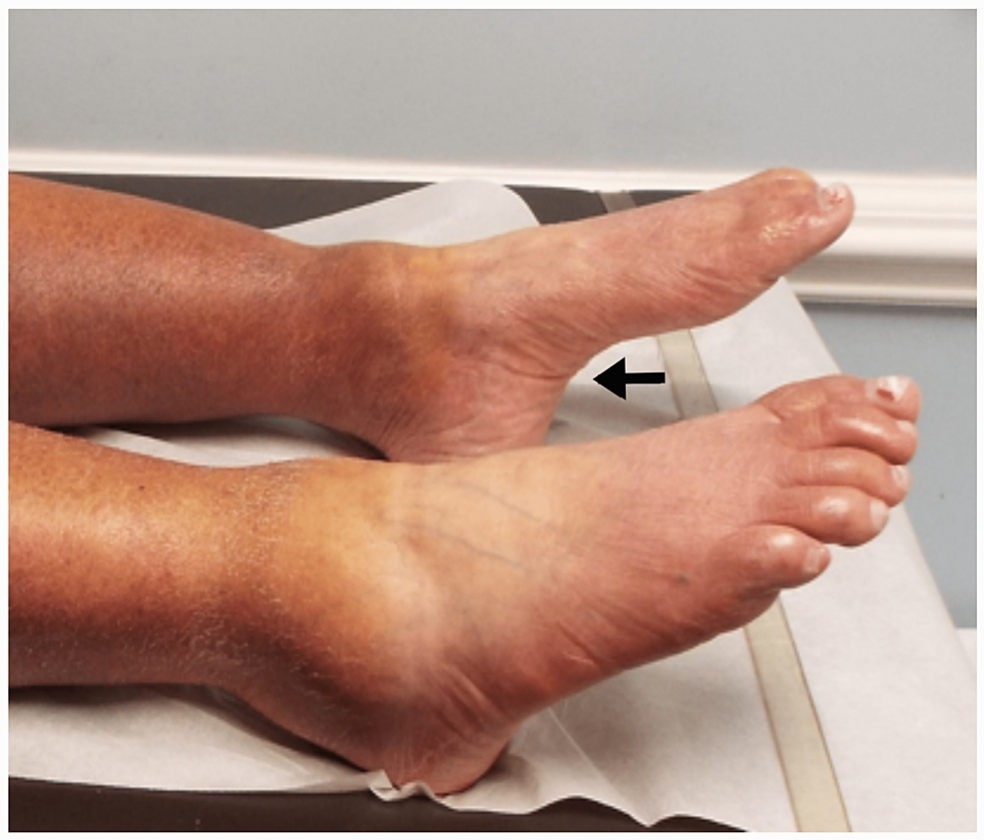
High-arched feet Patient 1: The arrow points to the high-arched foot. The patient was unable to dorsiflex either foot.

A 71-year-old female started experiencing numbness in her feet at age 39 and later began to trip and fall at age 50. When she was initially evaluated in our facility, she walked with an ankle foot orthosis (AFO) brace and a walker to help with ambulation and to avoid falls. Clinical findings are summarized in Table 1. High-arched feet were noted bilaterally (Figure 1).

**Table 1:** Clinical details of mother and her son R: Right; L: Left; B: Bilateral; H: Hand; For: Forearm; F: Foot; Le: Leg; Th: Thigh; APB: Abductor pollicis brevis; FDI: First dorsal interosseous; ADM: Abductor digiti minimi; UE: Upper extremities; LE: Lower extremities.

Metrics	Patient 1 (Mother): Age 65				Patient 1 (Mother): Age 71			Patient 2 (Son): Age 48	
Symptoms	Trips and falls, leg muscle weakness and atrophy, sensation loss in feet (15 yrs), hand paresthesia (1 yr)				Progressive worsening of weakness of lower and upper extremities			Pain/paresthesia of toes	
Ambulation	Walker				Wheelchair			Normal	
Cranial Nerves	Normal				Normal			Normal	
High-Arched Feet	Yes				Yes			Yes	
Tremor	Mainly the thumb (R > L)				B hands			None	
Muscle Strength	R		L		R	L		R	L
Upper Proximal	Normal		Normal		Normal	Normal		Normal	Normal
Upper Distal	Weak H		Weak H		Weak H & For	Weak H & For		Normal	Normal
Lower Proximal	Normal		Normal		Weak Quad	Weak Quad		Normal	Normal
Lower Distal	Weak F & Le		Weak F & Le		No movement	No movement		Normal	Normal
Muscle Atrophy		R	L		R	L		R	L
Upper		APB, FDI, ADM	APB, FDI, ADM		H & For	H & For		None	None
Lower		F & Le	F & Le		F, Le, Th	F, Le Th		None	None
Reflexes		R	L		R	L		R	L
Biceps		Diminished	Diminished		Absent	Absent		Normal	Normal
Triceps		Diminished	Diminished		Absent	Absent		Normal	Normal
Knee		Absent	Absent		Absent	Absent		Normal	Normal
Ankle		Absent	Absent		Absent	Absent		Absent	Absent
Sensory: UE		R	L		R	L		R	L
Loss of Pain		All digits	All digits		Up to elbow	Up to elbow		None	None
Loss of Touch		All digits	All digits		Up to elbow	Up to elbow		None	None
Loss of Position		None	None		None	None		None	None
Sensory: LE		R	L	R		L	R		L
Loss of Pain		Up to knee	Up to knee	Up to mid thigh		Up to mid thigh	Foot		Foot
Loss of Touch		Up to knee	Up to knee	Up to mid thigh		Up to mid thigh	None		None
Loss of Position		Big toe	Big toe	Toes & ankles		Toes & ankles	None		None
Loss of Vibration		Ankle	Ankle	Ankle & knee		Ankle & knee	None		None

**Figure 2:**
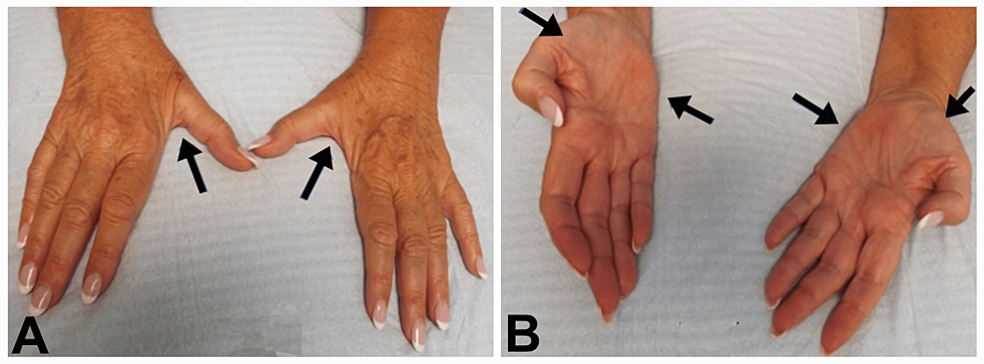
Bilateral wasting of the intrinsic hand muscles Patient 1: Bilateral wasting of the intrinsic hand muscles. (A) Arrows point to wasting of the first dorsal interosseous muscles bilaterally. (B) Arrows point to wasting of the thenar and hypothenar muscles bilaterally.

A significant weakness of the dorsiflexors and plantar flexors of the feet was noted bilaterally (dorsiflexor weakness was more pronounced) with loss of the ankle reflexes. Decreased pinprick and light touch sensations up to the midcalf and absent vibration sense at the ankle were observed. Position sense was erratic at the big toes. There was also the weakness of intrinsic hand muscles and decreased pain and light touch sensations over the digits and the hands up to the wrist. The patient was evaluated six years later at which time she had experienced significant progression of muscle weakness and needed a wheelchair. The knee and ankle reflexes were absent. Pinprick and light touch were absent up to the knees. There was an absence of both position sense in the big toes as well as vibration sense at the ankles and knees. The upper extremities showed significant weakness and wasting of the intrinsic hand muscles (Figure 2A, 2B), and loss of pinprick and light touch sensation had extended up to the elbows. Biceps and triceps DTRs were absent bilaterally. She had rest and postural tremors of the fingers, the right more than the left. Cranial nerves were normal. CMTNS was 29. 

During the six years following the initial encounter, there was a significant worsening in muscle strength and more atrophy. The thigh muscles were normal at the first visit but showed weakness and atrophy during the second visit. The biceps and triceps reflexes were intact (although diminished) at the initial visit but were absent at the subsequent visit. The level of sensory loss also progressed steadily to involve the more proximal areas of the upper and lower limbs (Table 1). 

EDX Studies

The nerve conduction and electromyography (EMG) findings are summarized in Table 2. Compound muscle action potentials (CMAPs) were absent on fibular and tibial nerve stimulation bilaterally. MNCV of the median and ulnar nerves was >38 m/s, and no sensory nerve action potentials (SNAPs) could be recorded over the superficial radial, median, ulnar, sural, and superficial fibular nerves. The patient could not recruit motor unit potentials in the tibialis anterior, while the gastrocnemius showed 1-2 large, long-duration polyphasic motor units. The distal upper extremity muscles showed a decrease in motor unit recruitment with an increase in polyphasic motor units. 

**Table 2:** Electrodiagnostic findings of mother and her son DL: Distal latency in milliseconds; Amp: Amplitude in millivolts for motor and in microvolts for sensory; CV: Conduction velocity (m/s); NR: Not recordable. Normal values: Median motor: DL ≤ 4.4 ms; Amp ≥ 4 mV; CV ≥ 49 m/s. Median sensory: DL ≤ 3.4 ms; Amp ≥ 25 uV. Ulnar motor: DL ≤ 3.5 ms; Amp ≥ 2 mV; CV ≥ 49 m/s. Ulnar sensory: DL ≤ 3.4 ms; Amp ≥ 10 uV. Radial motor: DL ≤ 3.6 ms; Amp ≥ 2 mV; CV ≥ 49 m/s. Sup radial sensory: DL ≤ 3.0 ms; Amp ≥ 15 uV. Fibular motor: DL ≤ 6.0 ms; Amp ≥ 2 mV; CV ≥ 40 m/s. Sup fibular: DL ≤ 4.0 ms; Amp ≥ 4 uV. Tibial motor: DL ≤ 6.0 ms; Amp ≥ 2mV; CV 40 m/s. Sural: DL ≤ 4.0 ms; Amp ≥ 10 uV.

Nerves	Patient 1 (Mother): Age 65			Patient 1 (Mother): Age 71			Patient 2 (Her Son): Age 48		
	DL	Amp	CV	DL	Amp	CV	DL	Amp	CV
L. Median Motor	5.6	2.6	42.5	5.6	0.56	39.2	4.0	6.8	41.5
R. Median Motor	5.1	3.7	39.0	5.1	1.58	41.6	3.9	6.6	40.6
L. Ulnar Motor	3.2	4.2	52.0	3.7	1.10	52.5	3.3	5.6	49.0
R. Ulnar Motor	3.2	4.5	55.0	4.3	0.94	55.2	3.0	5.9	50.0
R. Radial Motor	3.3	2.1	39.0	3.6	0.64	39.2			
R. Fibular Motor	NR			NR			4.2	0.96	30.0
L. Fibular Motor	NR			NR					
L. Tibial Motor	NR			NR			6.3	0.19	30.1
L. Median Sensory	NR			NR					
R. Med Sensory	NR			NR			4.1	17.0	
L. Ulnar Sensory	NR			NR					
R. Ulnar Sensory	3.7	5.8		4.2	3.8		3.3	9.8	
L. Sup Radial	NR			NR			NR		
R. Sup Radial	NR			NR			NR		
R. Sup Fibular	NR			NR			NR		
R. Sural	NR			NR			NR		

US Study

**Figure 3:**
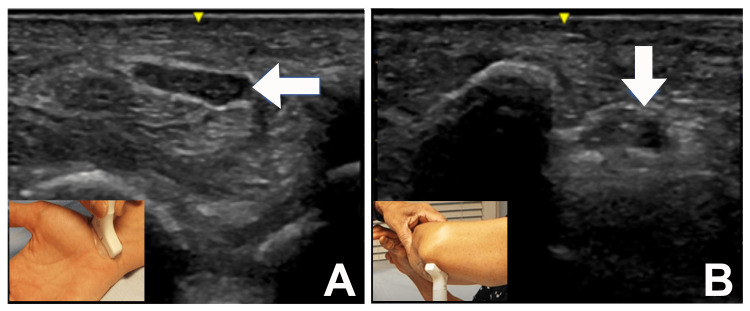
Ultrasound of the median nerve at the wrist and mid forearm Patient 1: Ultrasound of the median nerve at the wrist and mid forearm. (A) Short-axis view of the right median nerve at the carpal tunnel inlet (arrow). The cross-sectional area was 10 mm^2^ (normal: ≤12 mm^2^). (B) Short-axis view of the right ulnar nerve (arrow) at the elbow. The cross-sectional area was 9 mm^2^ (normal: ≤10 mm^2^).

The cross-sectional area (CSA) of the median nerve was measured at the wrist and mid-forearm (Figure 3A, 3B). The CSA of the median nerve was 10 mm^2^ at the wrist (normal: ≤12 mm^2^) and 7 mm^2^ at the mid-forearm (normal: ≤8 mm^2^). The ulnar nerve was also studied at the elbow, and the CSA was 9 mm^2^ (normal: ≤10 mm^2^).

Patient 2 (son)

Clinical Findings

A 48-year-old male reported a history of numbness and intermittent sharp pain in the feet bilaterally. He denied symptoms in the upper extremities and did not experience muscle weakness or a tendency for falls. Neurological examination showed high-arched feet. Pinprick sensation was diminished over the plantar and dorsal aspects of both feet. Position, vibration, and light touch sensations were normal. The ankle reflexes were absent bilaterally, and the knee reflexes were normal. Gait was also normal.

EDX Studies

The NCV/EMG studies showed slowing of the motor conduction of the median nerves, although were >38 m/s. The fibular nerves revealed slowing of motor conduction. No SNAPs could be recorded over the superficial fibular or superficial radial nerves; the median and ulnar nerve SNAPs showed a low amplitude. 

US Study

The CSA of the median nerve was measured at the wrist and mid-forearm. The CSA of the median nerve was 11 mm^2^ at the wrist (normal: ≤12 mm^2^) and 8 mm^2^ at the mid-forearm (normal: ≤8 mm^2^). The ulnar nerve was also studied at the elbow, and the CSA was 10 mm^2^ (normal: ≤10 mm^2^).

Family History

**Figure 4:**
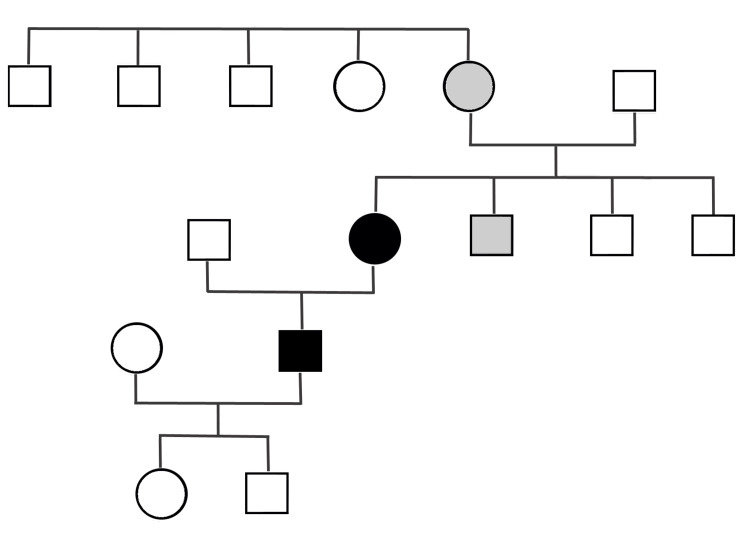
Pedigree chart of the family Dark: Circle: Patient 1; Square: Patient 2. Gray: History of walking problems (not evaluated).

No other family members have been diagnosed with CMT disease. Figure 4 depicts the pedigree chart of the family. The father of patient 1 had difficulty walking, but additional details could not be obtained. Her brother complained of numbness in the feet but refused to pursue a neurological evaluation or genetic testing.

Diagnosis of CMT Disease and Genetic Testing

Both patients were diagnosed with CMT2 disease based on clinical and EDX findings, with the latter test demonstrating the median nerve motor conduction velocity >38 m/s. Genetic studies confirmed a heterozygous identical variant of MPZ in Exon 2.c109G>A (p.Glu37Lys) for both patients 1 and 2. Inherited in an autosomal dominant fashion, this single-nucleotide variant has a cytogenetic location on chromosome 1q23.3 [7,12]. The variant was deemed to be of uncertain clinical significance by the lab as it has not been reported before. 

## Discussion

More than 300 *MPZ* gene mutations have been identified as the cause of inherited peripheral neuropathy, exhibiting a wide range of phenotypes with varying severity of clinical symptoms [7,9,13]. The early-onset infantile and childhood phenotypes developmentally impair myelination, while the adult-onset phenotypes are characterized by axonal degeneration without prior demyelination [7,14]. Sanmaneechai and colleagues provided detailed descriptions of phenotypes of MPZ mutations from a cohort of 103 patients from 71 families with 47 different mutations [15]. These authors categorized three phenotypes with different ages of onset and severity based on the mutations. The infantile group experienced developmental delay and difficulty with ambulation. This group had slow MNCV and absent SNAPs. The childhood-onset type also showed slow MNCV and decreased amplitude of SNAPs. The adult-onset group was the most common, showing a mild decrease in MNCV and a decreased amplitude of CMAPs. Callegari and colleagues studied the role of newly described mutations in late-onset phenotypes and classified the clinical pattern of *MPZ* gene mutations into early-onset, childhood/adolescent-onset, and late-onset phenotypes [16].

In Chen and colleagues’ study of genetic and clinical features in six patients with CMT1B from unrelated Chinese families, all patients initially complained of weakness in the lower extremities [7]. Six *MPZ* gene mutations were uncovered, including four known and two novel. EDX studies revealed a chronic progressive sensorimotor demyelinating polyneuropathy. These authors described that their patients with *MPZ* gene mutations had relatively mild clinical phenotypes [7]. Taniguchi and colleagues retrospectively studied 64 CMT patients with 23 previously reported *MPZ* gene variants and 21 patients with 17 novel *MPZ* gene variants [9]. A single Japanese patient exhibited the novel variant p.Glu37Lys (the same variant as in our patients). This patient presented at the age of 59 with complaints of severe weakness of the lower extremities without cranial nerve involvement. Symptom onset was at 39 years old. The median nerve MNCV was 55.9 m/s, and the CMT disease was classified as axonal. The amino acid change was p.Glu37Lys, and the nucleotide change was 109 G> A. An in silico analysis revealed the following: PROVEAN: -2.95, SIFT: 0, Polyphen 2: 0.992, and Mutation taster: disease-causing. According to the American College of Medical Genetics standard and guidelines, the pathogenicity is PM1, 2, PP1, 3. Inherited in an autosomal dominant manner, it was considered “likely pathogenic” as it fulfilled “2 categories of moderate pathogenic evidence and 2 supportive pathogenic evidence” [14]. The MPZ, c.109G>A (p.Glu37Lys) variant is absent in gnomAD. This suggests that the variant is rare. A similar phenotype was noted in variants p.Asp75Gly and p.Ser111Tyr that occurred at or near the same codon. These authors presumed that the confirmed novel variants likely induced a pathogenic phenotype, especially in these missense variants [9]. This particular MPZ genetic variant has not been reported in other countries. 

Two categories of MPZ mutations are responsible for a demyelinating phenotype: (1) nonsense and frameshift mutations that delete large segments of MPZ leading to loss of structure and function and (2) missense mutations that disturb the structure and function of MPZ, cause intracellular folding problems with MPZ, or mislocalize MPZ [8]. Missense mutations comprise amino acid alterations that result in lost or altered MPZ function [8]. 

The family described in this case report provided a unique opportunity to study the phenotypical features of the p.Glu37Lys *MPZ* gene mutation in CMT disease in early and late stages. Patient 1 started having symptoms at age 39 and demonstrated more advanced disease at her initial visit with upper extremity involvement. Patient 2 experienced sensory symptoms of the lower extremities at age 47. Both patients had sensory abnormalities of the distal lower extremities and absent ankle reflexes. The mother had significant weakness in the feet and intrinsic hand muscles. The MNCV of the median nerves was slowed in both patients, and SNAPs were absent over the superficial radial and superficial fibular nerves. Both patients’ findings were consistent with CMT2 disease with axonal involvement. 

The study of *MPZ* gene mutations and the resulting phenotypical features is crucial for understanding the pathogenetic mechanism. While demyelinating neuropathies can be easily explained based on the role of MPZ in myelin compaction, the mechanism of axonal neuropathies is not fully understood. One theory is that MPZ-related axonal neuropathies may be due to the disruption of myelin-axon interactions [14]. The occurrence of axonal neuropathy without significant demyelination (CMT2J) when methionine substitutes threonine at position 124 of PO (POT124M0) provides an excellent opportunity to explore the pathogenesis of axonal neuropathy. Shackleford and colleagues studied a mouse model of this genetic mutation and concluded that metabolic changes from this mutation can lead to axonal degeneration and prominent alterations in non-compact myelin domains such as paranodes and gap junctions [17]. Understanding the molecular and cellular pathogenesis with the advances in gene therapy can potentially lead to better treatment options [18]. The association of biallelic mutations in the sorbitol dehydrogenase gene in some patients with the axonal form of CMT disease is likely to respond to aldose reductase inhibitors that are already available [19].

## Conclusions

To our knowledge, only one previous patient has been reported with the newly described MPZ variant p.Glu37Lys in CMT disease. Our two patients with this same MPZ variant offer valuable insight into the progression of the disease from the early to the late stages of CMT disease. We also illustrate the clinical, EDX, and US features of the newly described mutation of MPZ in CMT disease. 
